# Neural Tracking to Auditory Statistical Structures in Children

**DOI:** 10.1002/pchj.814

**Published:** 2024-11-30

**Authors:** Zihe Zhang, Lingzhi Kong

**Affiliations:** ^1^ Language Pathology and Brain Science MEG Laboratory, School of Communication Sciences Beijing Language and Culture University Beijing People's Republic of China

**Keywords:** auditory, children, entrainment, magnetoencephalography, statistical learning

## Abstract

Children's brain is able to track the linguistic structures in continuous speech. When there was no prior knowledge, we found that children also automatically detected and tracked the statistical structures in auditory tone steam as reflected by neural entrainment, but their ability was immature.

Adult brain is able to track linguistic structures in speech, for example, syllables and phrases (Ding et al. 2016, 2018). Using frequency‐tagging paradigm, we have found that children's brain could track the frequency (4 Hz) of syllables, but their neural tracking to higher‐level language units, that is, phrase, was immature compared to that of adults (Kong et al. 2023). One question raised here is whether children's brain is also immature in tracking the higher‐level structures in auditory stream without prior linguistic knowledge.

In order to control the impact of linguistic knowledge, the current study adopted one of the research paradigms about statistical learning, using auditory tone stream with embedded statistical structures (triplet) as stimuli to test if children can track the frequency of the triplet (1 Hz). Previous study have found that adult brain performed robust neural entrainment to the frequency of triplet during exposure to the auditory tone stream (Moser et al. 2021). To our knowledge, there is no research on the neural tracking of auditory statistical tone structures (triplet) in children. According to the behavioral evidence that nonlinguistic auditory statistical learning improved during childhood (Shufaniya and Arnon 2018), we hypothesized that the neural tracking of the auditory statistical structures in children would be weaker than that of adults.

We recruited 17 children (6 males; aged 4.17–8.00 years; mean age = 6.12 ± 1.32 years) and 16 young adults (1 males; aged 18.42–26.50 years; mean age = 22.79 ± 2.44 years). Their native language is Mandarin.

Each tone sequence consisted of 12 pure tones whose frequencies were between 263.63 and 932.33 Hz. The frequency of each tone corresponded to notes C, D, E, F#, G#, and A# from the fourth and fifth octave of a standard piano (Moser et al. 2021). The 12 tones in one sequence were grouped into triplets consisting of three tones within one octave. There were two different types of tone sequences: the structured condition and the random condition. For the structured condition, the tone sequence comprised only four types of triplets that repeated in a random order over the course of sound exposure, leading to a structured tone stream (see Figure 1A). The inner order in each triplet was counterbalanced across participants. For the random condition, the three tones in each triplet varied throughout the sound exposure, resulting in a pseudo‐random tone stream. Thus, the random stream and the structured stream consisted of same amount of tones while the structured stream contained an embedded statistical pattern of repeated triplets.

**FIGURE 1:**
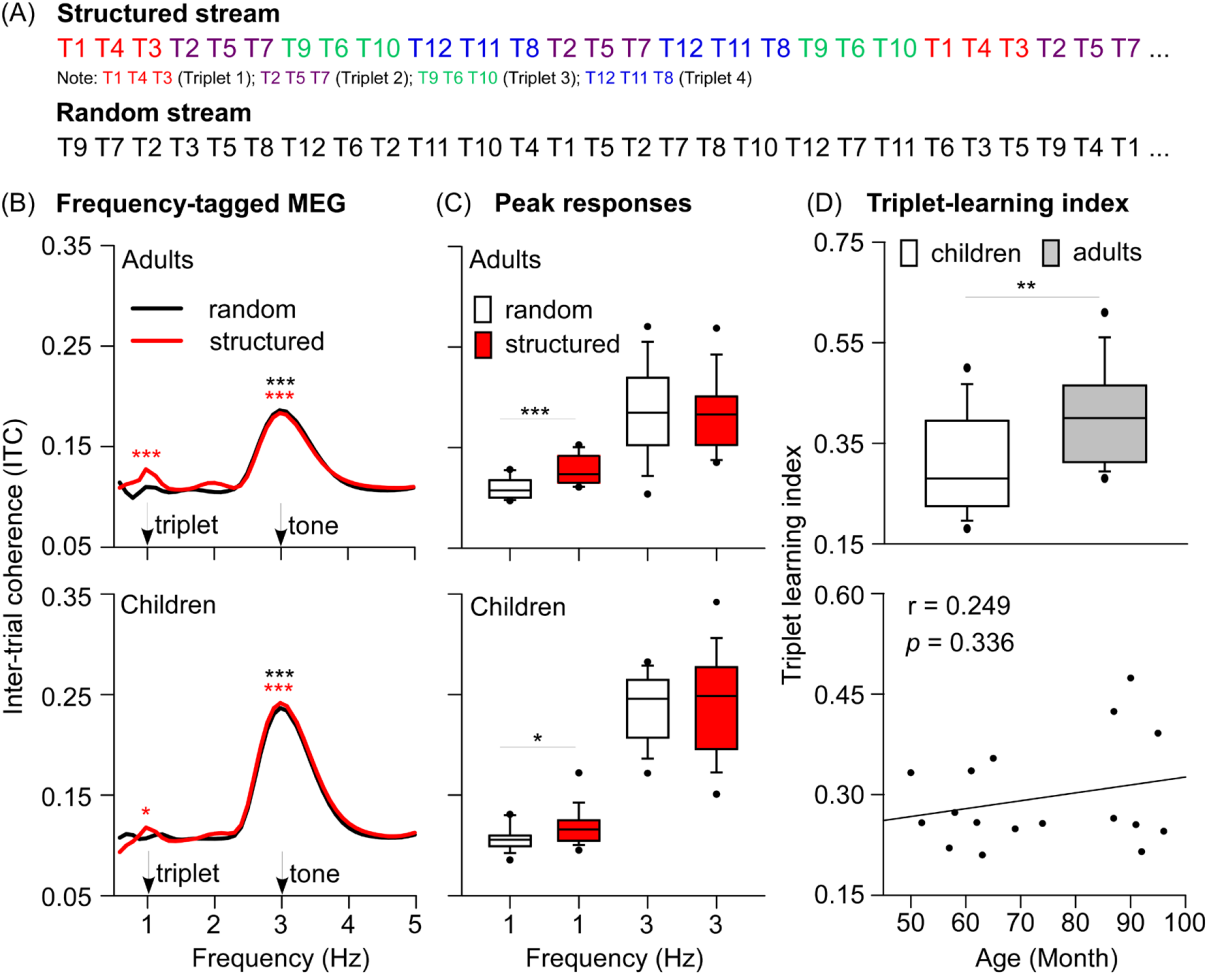
(A) Demonstration of the stimuli. (B) Frequency‐tagged MEG. Comparison of ITC at triplet and tone frequencies with the mean ITC of the neighboring eight frequency bins. (C) Peak responses. Comparison of ITC at triplet and tone frequencies in the structured condition with those of the random condition. (D) Triplet‐learning index. Comparison of triplet‐learning index between children and adults (higher panel) and the correlation between triplet‐learning index and age (lower panel). *p < 0.05; **p < 0.01; ***p < 0.001.

For both conditions, each stream consisted of 2520 tones (840 triplets). The duration of each tone was 300 ms and the tones was delivered every 333 ms with an inter‐tone interval of 33 ms. Thus, the individual tones occurred in a frequency of 3 Hz and the triplet occurred in a frequency of 1 Hz. During exposure, participants first listened to the random stream, then the structured stream, in order to avoid applying knowledge and expectations acquired during the structured block to the random block. While listening to the tone stream, participants watched a silent cartoon movie (The Little Prince).

Magnetoencephalography (MEG) data were collected by a 64‐channel Yokogawa MEG system (Yokogawa/KIT, Kanazawa, Japan) with a sampling rate of 1000 Hz. Auditory stimuli were delivered via air‐conducted earphones at a comfortable level and kept the same across subjects. MEG data were filtered through the 0.1–30 Hz bandpass filter. Upon visual inspection, no channel with high‐level noise was identified in all 64 channels. Then data was time‐locked to the onset of each triplet and extracted into 70 epochs. Signals from all MEG channels were averaged to index the whole‐brain response. We computed the strength of neural entrainment across frequencies by computing inter‐trial coherence (ITC) across epochs, using a fast Fourier transform with Hanning windows. Then we calculated the mean triplet‐learning index (ITC_triplet_/ITC_tone_) in adults' and children's groups. If listeners were more sensitive to the triplets, there would be a more significant preferential shift in the neural entrainment from individual tones to underlying triplet structure, resulting in a higher triplet‐learning index (Batterink and Paller 2017).

One‐tailed paired *t*‐test was adopted to examine whether the ITC was significant at the tagged frequencies (1 and 3 Hz) compared to the average of the two neighboring bins (Kong et al. 2023). The independent *t*‐test was performed to examine the effect of condition and age group on the strength of neural tracking. The Pearson correlation analysis examined the effect of age on triplet‐learning index of children.

During the exposure, both children and adults performed robust neural entrainment at triplet frequency (1 Hz) (children: *p* = 0.019, adults: *p* < 0.001) and tone frequency (3 Hz) (children: *p* < 0.001, adults: *p* < 0.001) in the structured block. In contrast, both two groups only performed significant neural entrainment at tone frequency (children: *p* < 0.001, adults: *p* < 0.001) in the random block (see Figure 1B). For both children and adult, ITC at 1 Hz was significantly higher in the structured condition compared to random condition (children: *p* = 0.020, adults: *p* < 0.001), while no significant difference in ITC was observed at 3 Hz between the two conditions (children: *p* = 0.471, adults: *p* = 0.568) (see Figure 1C). Thus, our result showed that both children and adults automatically detected and tracked the auditory statistical structures (triplet).

Figure 1D showed the triplet‐learning index of children and adults. The adults' triplet‐learning index was significantly higher than that of children (*p* = 0.009), which indicated that children's sensitivity to auditory statistical structures was less pronounced than that of adults. There are two possible reasons: children's inferior statistical learning and the immaturity of children's neural tracking to higher‐level structures in the auditory stream. However, the triplet‐learning index was not significantly correlated with children's age (*r* = 0.249, *p* = 0.336), despite an increasing tendency of triplet‐learning index with age (see Figure 1D, lower panel).

In summary, we found that children's brain was able to detect and track the auditory statistical structures in tone stream when they had no prior knowledge of the structures. Moreover, children's neural tracking to the statistical structures might be weaker than that of adults. As statistical learning is believed to play an important role in language acquisition, our findings suggested that children's brain is able to build higher‐level language structures based on statistical cues while this ability is weaker than adults.

## Ethics Statement

This study was approved by the Human Research Ethics Committee in Beijing Language and Culture University.

## Conflicts of Interest

The authors declare no conflicts of interest.
